# Integrated bioinformatics and tissue-based validation reveal the oncogenic role of hsa_circ_0043256 and hsa_circ_0004789 in gastric cancer

**DOI:** 10.1016/j.bbrep.2026.102442

**Published:** 2026-01-09

**Authors:** Somayeh Aslani, Ashkan Kalantary-Charvadeh, Pejman Morovat, Roghayeh Abbasalipourkabir, Issa Nourmohammadi, Amirnader Emami Razavi, Nasrin Ziamajidi

**Affiliations:** a Department of Clinical Biochemistry, School of Medicine, Hamadan University of Medical Sciences, Hamadan, Iran; b Student Research Committee, Hamadan University of Medical Sciences, Hamadan, Iran; c Department of Quality Control, Razi Vaccine and Serum Research Institute, Agricultural Research, Education and Extension Organization (AREEO), Karaj, Iran; d Department of Chemistry, University of Central Oklahoma, Edmond, OK, United States; e Iran National Tumor Bank, Cancer Institute of Iran, Tehran University of Medical Sciences, Tehran, Iran; f Research Center for Molecular Medicine, Institute of Cancer, Avicenna Health Research Institute, Hamadan University of Medical Sciences, Hamadan, Iran

**Keywords:** Stomach neoplasms, Circular RNA, MicroRNA, Cyclins, Computational biology

## Abstract

Circular RNAs (circRNAs) play a key role in gastric cancer (GC) pathogenesis. This study hsa_circ_0043256 and hsa_circ_0004789, and their interactions with miR-28–5p/Cyclin B1 (CCNB1) and miR-5683/CCNB1, via bioinformatic and experimental methods. We retrieved expression data for circRNAs, miRNAs, and mRNAs in GC from Gene Expression Omnibus and The Cancer Genome Atlas. Using online databases and R tools, we identified downstream miRNAs and target mRNAs to build a competing endogenous RNA (ceRNA) network. After identification of hub genes and performing functional enrichment, we defined two regulatory axes: hsa_circ_0043256/miR-28–5p/CCNB1 and hsa_circ_0004789/miR-5683/CCNB1. We studied 32 paired tumor and adjacent tissues to assess all genes and CCNB1 protein expression, along with correlations, histopathological associations, ROC curves, and survival outcomes. We identified 58 circRNAs, 123 miRNAs, and 2126 mRNAs, and, further, by novelty checking and downstream RNA analysis, identified two axes. The expression of hsa_circ_0043256, hsa_circ_0004789, and CCNB1 mRNA and protein levels was elevated, while miR-28–5p and miR-5683 levels were reduced. Correlations observed among axis components supported the ceRNA hypothesis. The hsa_circ_0004789/miR-5683/*CCNB1* axis showed an AUC value for the combined ROC curve near 1, suggesting strong diagnostic potential. Lower *CCNB1* and higher miR-5683 levels were correlated with better survival. Both circ_0043256 and circ_0004789 were associated with histological grade, lymphatic invasion, perineural invasion, and lymph node involvement. This study highlights circ_0043256/miR-28–5p/CCNB1 and circ_0004789/miR-5683/CCNB1 as promising axes for GC diagnosis and treatment strategies.

## Highlights

• Upregulated hsa_circ_0043256 and hsa_circ_0004789 in GC tissues correlate positively with CCNB1 and inversely with hsa-miR-28–5p and hsa-miR-5683, respectively.
• The combined ROC curve for hsa_circ_0004789/miR-5683/CCNB1 axis was close to 1, indicating its potential for GC diagnosis.
• Hsa_circ_0043256 and hsa_circ_0004789 associate with histopathological features such as grade, lymphatic/perineural invasion and lymph node involvement.
• Hsa_circ_0043256 and hsa_circ_0004789 show promise as GC biomarkers and therapeutic targets.

## Graphical abstract

(**A**) A schematic representation of the proposed mechanism, where hsa_circ_0043256 and hsa_circ_0004789 act as sponges for hsa-miR-28–5p and hsa-miR-5683, preventing their access to *CCNB1* mRNA. As a result, *CCNB1* mRNA is not degraded and is translated, leading to cell cycle progression. (**B**) The lower part of the figure schematically shows all the study steps. Bioinformatics analysis suggested the circ_0043256/miR-28–5p/CCNB1 and circ_0004789/miR-5683/CCNB1 axes. This study included 32 pairs of tumor and margin tissues. RNA was extracted and gene expression analysis was done by RT-qPCR. The level of CCNB1 protein was evaluated by Western blot after protein extraction.

**Figure:**
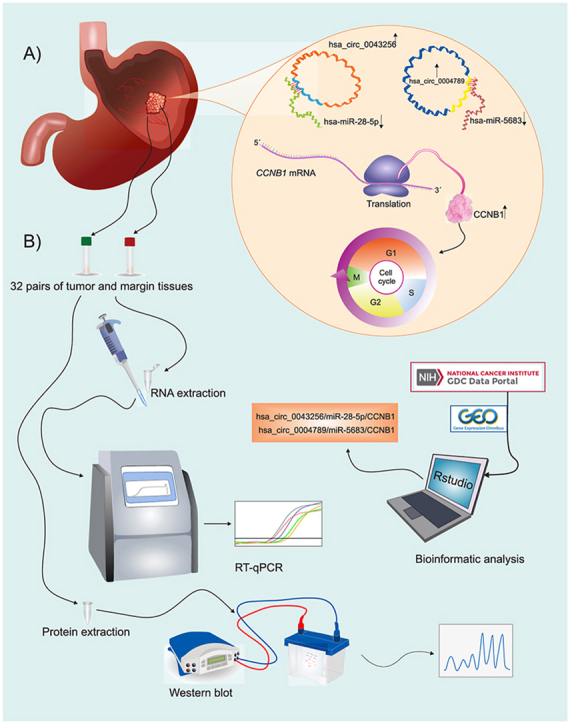

## 1 Introduction

Gastric cancer (GC) is the fifth most common cancer and the third in terms of death is caused by cancer in the world. Every year, about 990,000 patients with GC are diagnosed worldwide, and about 738,000 of them die. Poor prognosis, diagnosis in advanced stages, as well as limited treatment options, increase the death rate from GC. Investigating the cellular mechanisms of GC carcinogenesis is critical for identifying specific diagnostic biomarkers and new treatment strategies [1]. Non-coding RNAs (ncRNAs) are regulators of cellular mechanisms that do not encode proteins but play roles in many cellular processes, such as transcription, splicing, and translation [2]. A group of ncRNAs is microRNAs (miRNAs), which are 19–25 nucleotides in length and act as post-transcriptional regulators [3]. miRNAs bind target mRNAs, leading to their degradation or translation inhibition [4]. miRNAs are expressed in different tissues, and their presence in body fluids has been proven. miRNAs are effective at multiple levels, from initiation to progression, in many cancers, including GC [5]. Circular RNAs (circRNAs) are a newly recognized group of ncRNAs that have a very interesting structural feature: they form a closed loop with no free ends. This special structure gives them stability against exonuclease enzymes. Therefore, circRNAs have a special advantage as non-invasive biomarkers for various diseases, including cancer [6,7]. High-throughput studies have revealed the expression of distinct circRNAs across various tissues and their dysregulation in numerous diseases [8]. The study of these ncRNAs can help understand the pathogenesis of the disease and find new, effective treatment methods [6]. The regulatory potential of circRNAs has not yet been precisely elucidated. The most reported function of circRNAs is miRNA sponging, which occurs through the miRNA response element (MRE) sequence.

The “competing endogenous RNA” (ceRNA) hypothesis has been proposed to explain the interactions between ncRNAs. According to this hypothesis, a portion of transcription products forms a vast regulatory network across the transcriptome, including miRNAs, long non-coding RNAs (lncRNAs), pseudogenic RNAs, and circRNAs. These transcripts perform their regulatory function by competing for miRNAs; thus, the upstream ncRNA, by targeting the downstream miRNA, regulates its target mRNA. Therefore, a regulatory axis is formed by upstream ncRNAs, miRNAs, and target mRNAs [9]. Dysregulation of ceRNA network components is linked to cancer initiation and progression [10]. For example, circPVT1 affects cell growth, invasion, and migration in GC by regulating the miR-423–5p/Smad3 axis [11].

**Fig. 1:**
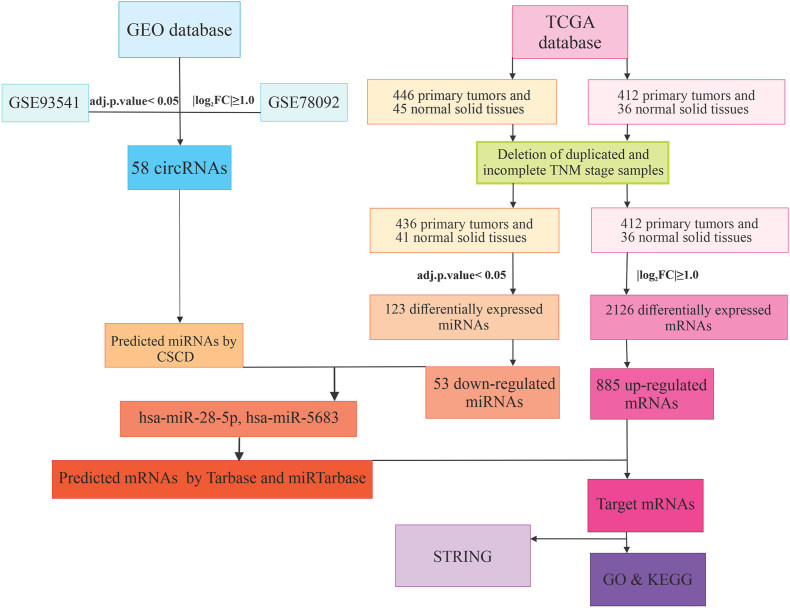
Flowchart illustrating the sequential steps and methods used in the bioinformatics analysis.

This research aimed to identify novel dysregulated circRNAs in GC through bioinformatics analysis, demonstrating that these circRNAs act as ceRNAs to modulate downstream miRNAs and play an essential role in regulating target mRNA expression. The flowchart for this study is shown in Fig. 1. In this study, the Gene Expression Omnibus (GEO) database was used to obtain circRNA microarray data; mRNA and miRNA sequencing data were obtained from the Cancer Genome Atlas (TCGA) database. Using R, the analysis identified differentially expressed circRNAs (DECs), miRNAs (DEMIs), and mRNAs (DEGs). By using the R language software and other online tools, DECs-related miRNAs and target mRNAs were predicted. In the next step, the protein-protein interaction network was formed by using STRING, an online search tool, and hub genes were identified. Finally, functional and pathway enrichment analyses were performed through the GO and KEGG databases. In this study, 32 pairs of tumor and margin tissues were used. Total RNA was extracted, and gene expression was measured in tumor samples compared to the margin. Western blot was used to measure Cyclin B1 (CCNB1) protein levels. Finally, the correlation between gene expression, ROC curve analysis, survival analysis, and the examination of the correlation between clinicopathological features and gene expression were analyzed using statistical methods.

## 2 Methods and materials

### 2.1 Bioinformatic methods

#### 2.1.1 Extraction of differentially expressed circRNAs (DECs) from microarray data

In order to find DECs in GC, microarray datasets providing the circRNA expression profile of the GEO database (https://www.ncbi.nlm.nih.gov/geo/) were used. Two datasets were downloaded from GEO using the GEOquery package in R (V 4.1.0). These two datasets include GSE93541 and GSE78092, with the gene chip platform for GSE93541 being Agilent-069978 Arraystar Human CircRNA microarray V1, and for GSE78092, ArrayStar Human Circular RNA microarray V2.0. The GSE93541 dataset includes three plasma samples from GC patients and three healthy controls, and the GSE78092 dataset contains three paired GC and adjacent normal mucosa tissues. The raw data were checked for normality and did not require normalization and log 2 transformation. Using the SVA package [12], two datasets were merged, and hidden batch effects were removed. The SVA package utilizes the ComBat function to adjust for batch effects. This empirical Bayesian approach is widely recognized for its effectiveness in mitigating batch effects in microarray data [13]. In the next step, the R limma package [14] was used to detect DECs using the statistical significance criterion |log2FoldChange| ≥ 1.0 and adj. p-value <0.05.

#### 2.1.2 Extraction of differentially expressed mRNAs (DEGs) and differentially expressed miRNAs (DEMIs)

To extract differentially expressed mRNAs (DEGs) and differentially expressed miRNAs (DEMIs), we obtained RNA and miRNA sequencing expression profile data, along with clinical data, from the GDC data portal of TCGA (https://portal.gdc.cancer.gov/). In the TCGA-STAD project for GC data, the number of RNA samples included 412 primary tumors and 36 normal solid tissues, while the numbers of miRNA samples were 446 and 45, respectively. Duplicate samples and samples with unknown TNM stage were removed. So, the number of tumor and normal RNA samples was reduced to 412 and 36, respectively, and for miRNA, the tumor and normal sample counts were reduced to 436 and 41, respectively.

Raw data of RNA-Seq and miRNA-Seq were normalized based on VOOM [15] method by the R package GDCRNATools [16]. To gain DEGs and DEMIs, expression data analysis for miRNA and RNA was done with DESeq2 package [17] between primary tumors and normal solid tissues. This analysis was performed based on the cutoff criteria of |log_2_FoldChange| ≥ 1 and adj. p-value <0.05, that was statistically approved.

#### 2.1.3 The prediction of circRNA-miRNA interaction

miRNA response elements (MREs) are physical communication rings between DECs and DEMIs. To predict MREs, first, the chromosomal location related to each DECs was extracted from the Circbase database (http://www.circbase.org/). Next, based on the MREs' location of DECs, the Cancer-Specific CircRNA Database (CSCD, http://gb.whu.edu.cn/CSCD/) was used to extract DEC-related miRNAs. CSCD-predicted miRNAs targeted by DECs were named CSCDmiRs. To extract target miRNAs corresponding to oncogenic DECs, an intersection was performed between CSCDmiRs and downregulated DEMIs, which were obtained from TCGA, using the cutoff criteria of log2FoldChange < −1 and adj. p-value <0.05. These miRNAs are referred to as final intersected miRNAs (FImiRNAs). Additionally, to identify tumor suppressor DEC-related miRNAs, an intersection was done between CSCDmiRs and TCGA-derived up-regulated DEMIs (log2FoldChange ≥1 and adj. p-value <0.05).

#### 2.1.4 Prediction of miRNA–mRNA interaction

To identify target mRNAs, the multiMiR package [18] in R was used to search multiple miRNA databases and predict potential target mRNAs for miRNAs, which we call MulmRNAs. The multiMiR package (http://multimir.org/) provides target mRNAs for miRNAs using validated databases (miRTarBase and TarBase) and predicted databases. Then an intersection was performed between MulmRNAs and TCGA, resulting in upregulated DEGs with cutoffs of log2FoldChange ≥1 and adj. p-value <0.05. This resulted in the identification of final intersected mRNAs (FImRNAs) as tumor-suppressor DEMI-related mRNAs. Conversely, the intersection of MulmRNAs and TCGA-derived down-regulated DEGs yielded oncogenic DEMIs-related mRNAs.

#### 2.1.5 Formation the regulatory network of circRNA/miRNA/mRNA

The regulatory network of circRNA/miRNA/mRNA was constructed based on previous results and predictions. According to the ceRNA hypothesis, the predicted interactions of DECs-FImiRNAs and FImiRNAs-FImRNAs formed a regulatory network of circRNA/miRNA/mRNA, which was visualized using Cytoscape software (V 3.10.2), a robust tool for illustrating biological networks.

#### 2.1.6 Construction of protein–protein interaction (PPI) network and study of hub genes

Protein–protein interaction (PPI) networks of FImRNAs were generated using the STRING database (https://string-db.org/) [19]. PPI analysis and visualization were performed using Cytoscape, with an interaction score >0.4. Hub genes were selected by the CytoHubba plugin based on the Maximal Clique Centrality score (MCC) [20].

#### 2.1.7 Functional enrichment analysis

To identify the enriched pathways and functions of the extracted top FImRNAs, we used the Gene Ontology (GO) [21] and Kyoto Encyclopedia of Genes and Genomes (KEGG) [22] databases.

The GO analysis was done in three areas: biological process (BP), cellular component (CC), and molecular function (MF). Analysis and visualization were performed using the ClusterProfiler R software package [23]. The GO and KEGG analyses were performed according to the Benjamini–Hochberg method, with an adjusted p-value threshold of <0.05.

### 2.2 Experimental methods

#### 2.2.1 Patients and tissue specimens

This study was conducted in accordance with the Helsinki Declaration. All the protocols of this study were approved by the Ethics Committee of Hamadan University of Medical Sciences, Iran (Ethical code: IR.UMSHA.REC.1401.749). Thirty-two paired fresh-frozen GC and adjacent normal tissues of GC patients were included in this study. The pathologist verified that all tumor and adjacent non-tumor tissues were identified as gastric cancer and margin tissues, respectively. Patients with a prior history of gastric cancer, other malignancies in different parts of the body, chemotherapy, radiotherapy, inflammatory diseases, or samples with recurrence were excluded. The tissues were transferred to liquid nitrogen immediately after surgery and preserved under proper conditions. Tissues were obtained from the Iran National Tumor Bank, Cancer Institute (Tehran University of Medical Sciences, Tehran, Iran). A report detailing all histopathological features of individuals who underwent surgery was provided to us.

#### 2.2.2 Genes expression analysis

Total RNA was extracted with TRIzol reagent (Ambion, Life Technologies, USA) based on the manufacturer's instructions. Extracted RNAs were reverse transcribed to cDNAs by Easy™ cDNA Synthesis Kit (Parstous, Iran) (for all genes except miRNAs). BON Stem High Sensitivity MicroRNA 1st Strand cDNA Synthesis Kit (BON Stem, Iran) was used for miRNA cDNA synthesis. RT-qPCR was performed using RealQ Plus 2 × Master Mix Green (Ampliqon, Denmark) by Roche Light Cycler 96 System (Roche Life Science, Germany). All of the primers listed in Supplementary Table 1. The expression results of *CCNB1* and circRNAs were normalized with the β-Actin gene, while miRNAs were normalized by U6. Data were analyzed with the 2^−ΔΔCt^ method.

#### 2.2.3 Western blot analysis

For protein extraction, tumor and margin tissues were powdered under liquid nitrogen and lysed with RIPA buffer (Kiazist, Iran) containing protease inhibitor cocktail. Extracted proteins were separated by sodium dodecyl sulfate polyacrylamide gel electrophoresis and transferred to a nitrocellulose membrane. After blocking with 5 % nonfat skimmed milk, the membrane was incubated overnight with primary antibody against CCNB1 (Santa Cruz, sc-245) and β-Actin (Santa Cruz, sc-69879), then with secondary antibodies at ambient temperature for 1 h. β-Actin was used as an internal control for normalizing CCNB1 protein expression. Image J software (version 1.41) was used for densitometry analysis of the protein bands.

#### 2.2.4 Statistical analysis

Statistical analysis was performed using both Prism 8.0.2 (GraphPad Software, La Jolla, CA) and SPSS version 26 (IBM SPSS Statistics; Chicago, IL, USA). The expression differences between tumor and margin tissues were analyzed using the Mann–Whitney U test for non-normal distributions and the independent t-test for normal distributions. The Pearson or Spearman correlation analysis was performed to investigate the correlation between gene expression based on normal or non-normal distributions, respectively. The diagnostic value of all genes was evaluated by drawing ROC curves. The correlation between gene expression and clinicopathological features was evaluated using the chi-square test. Moreover, the Kaplan-Meier method was used for survival analysis. All data were shown as mean ± SD, and p-values <0.05 were considered statistically significant.

## 3 Results

### 3.1 Identification of DECs, DEMIs, and DEGs

**Fig. 2:**
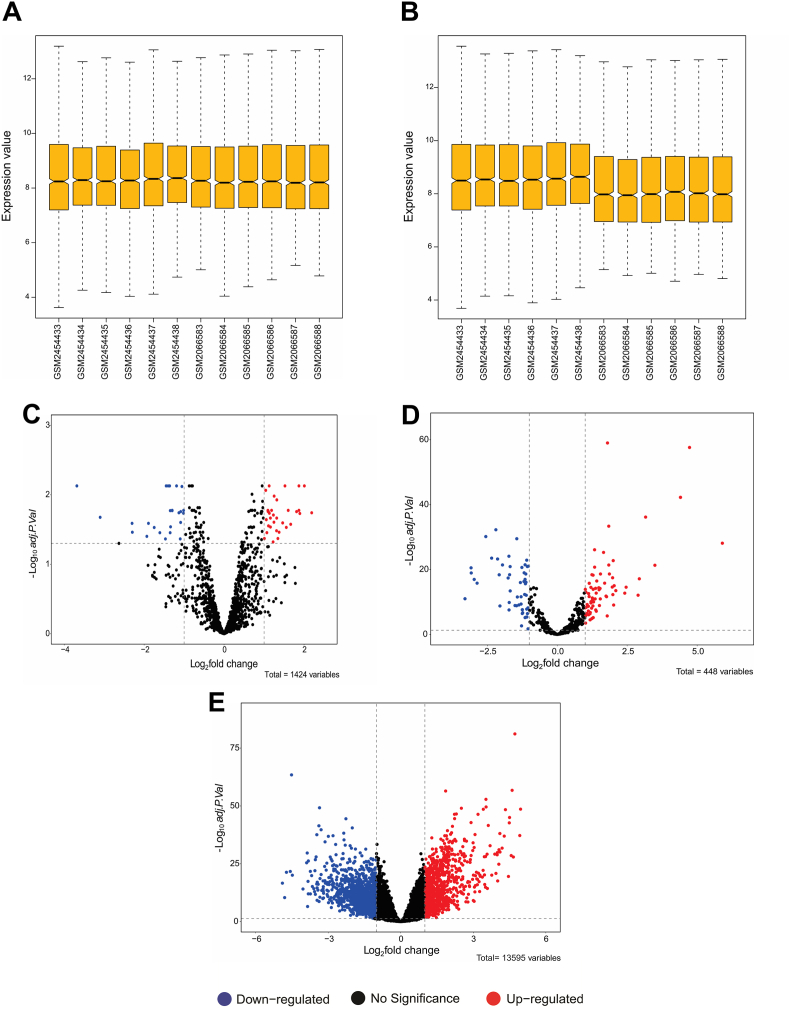
The box plots demonstrate the overall expression profiles of the two datasets: (**A**) before removing batch effect, and (**B**) after removing the batch effect. Volcano plots for (**C**) differentially expressed circRNAs (DECs), (**D**) differentially expressed miRNAs (DEMIs), and (**E**) differentially expressed mRNAs (DEGs).

The first step in regulatory network formation is the extraction of DECs. In this study, two microarray datasets of GC patients were analyzed. Two datasets were merged, and hidden batch effects were removed using the SVA package (Fig. 2A and B). Differential analysis was done by the R limma package, resulting in the identification of 58 differentially expressed circRNAs (Supplementary Table 2), with 33 upregulated and 25 downregulated circRNAs (Fig. 2C). Then, based on a literature review, we selected circRNAs that have not been studied in GC but have been investigated in other cancers for further analysis. Following the analysis of circRNAs' novelty and the identification of downstream miRNAs and mRNAs, we focused on circ_0043256 and circ_0004789. Although these circRNAs have not been previously explored in GC, they are significant players in other cancer types. The selected circRNAs, along with their essential features, are listed in Table 1.

**Table 1:** The key features of hsa_circ_0043256 and hsa_circ_0004789.

circBase ID	Alias	circBank ID	Host gene symbol	Position	log2FoldChange	adj.p.Value	Length
hsa_circ_0043256	hsa_circRNA_102046	hsa_circACACA_033	ACACA	chr17: 35604934–35609962 strand: -	1.81	0.01756757	483
hsa_circ_0004789	hsa_circRNA_102171	hsa_circSMURF2_006	SMURF2	chr17: 62587201–62594608 strand -	1.15	0.021635353	309

The next step in constructing the circRNA/miRNA/mRNA regulatory network was the identification of DEMIs and DEGs. The TCGA-STAD samples were analyzed with DESeq2 in R to compare primary tumor and normal solid tissue. DEMIs included 71 upregulated and 52 downregulated miRNAs (Fig. 2D). Also, DEGs included 885 upregulated and 1241 downregulated genes (Fig. 2E). The criteria for DEGs and DEMIs analysis were |log2FoldChange| ≥ 1 and adj. p-value <0.05.

### 3.2 Extraction of MREs and their matching target miRNAs

**Fig. 3:**
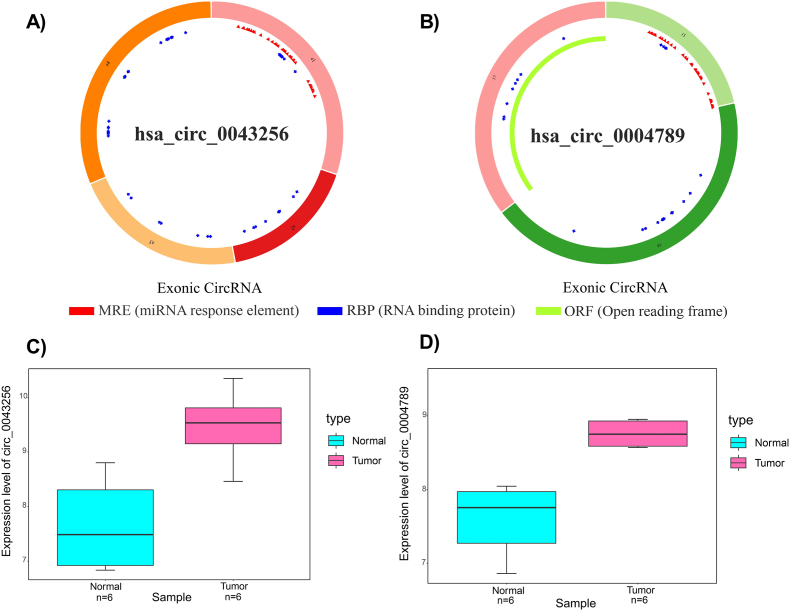
The schematic representations of (**A**) hsa_circ_0043256 and (**B**) hsa_circ_0004789 were obtained from the CSCD database. Box plots show the expression levels of (**C**) hsa_circ_0043256 and (**D**) hsa_circ_0004789 in both tumor and normal samples.

To predict target miRNAs, the MRE region of DECs was first determined using the CSCD database. The structure of circ_0043256, circ_0004789, and their expression levels in tumor and normal samples are shown in Fig. 3A–D. CSCD also provides a list of potential target miRNAs for each circRNA. Based on the ceRNA hypothesis and upregulation of selected DECs, an intersection was performed between CSCDmiRs and 52 downregulated DEMIs. The resulting miRNAs were called final intersected miRs (FImiRs) (miR-28–5p for circ_0043256, miR-5683, and miR-145–3p for circ_0004789), as shown in Table 2.

**Table 2:** Target miRNAs of circRNAs and their characteristics.

CircRNA	miRNA	log_2_FoldChange	adj.p.Value
hsa_circ_0043256	hsa-miR-28–5p	−1.051304797	2.7e-22
hsa_circ_0004789	hsa-miR-145–3p	−2.1478851	1.96e-24
	hsa-miR-5683	−2.964857723	1.26e-17

### 3.3 Prediction of target mRNAs of FImiRs and formation of ceRNA network

**Fig. 4:**
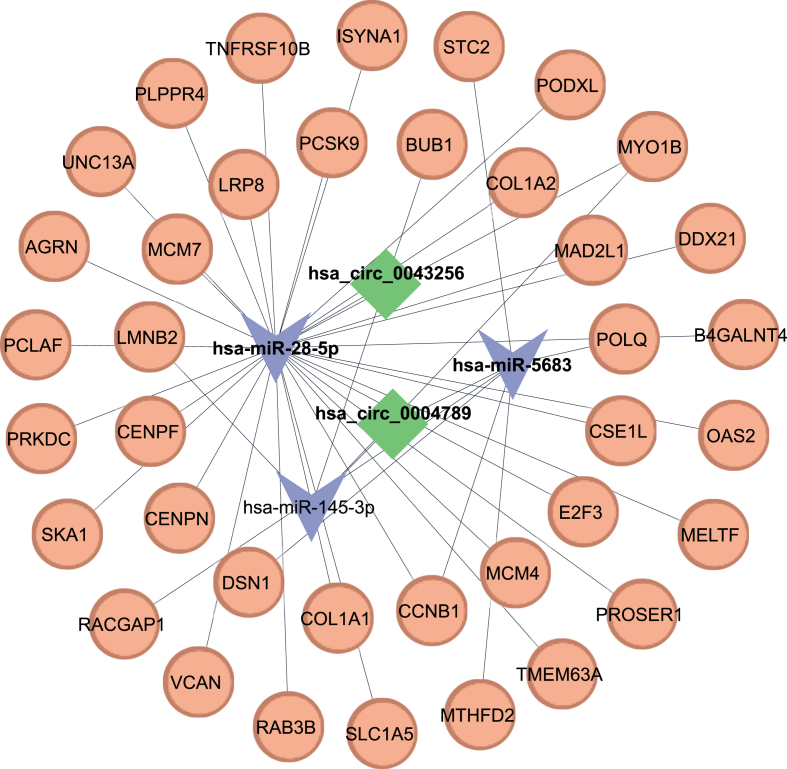
ceRNA network. The network consisting of two DECs (hsa_circ_0043256 and hsa_circ_0004789), three FImiRNAs (hsa-miR-28–5p, hsa-miR-5683, and hsa-miR-145–3p), and 38 FImRNAs. Diamond represents circRNA, V represents miRNA, and Ellipse represents mRNA.

Initially, the target mRNAs of FImiRs were determined using the multiMiR package in R, which searched multiple databases. This package predicted target mRNAs for miR-28–5p from the Tarbase database and for miR-145–3p and miR-5683 from the MiRTarbase database. Interestingly, the target mRNAs proposed by Tarbase and MiRTarbase have been experimentally confirmed. The final intersected mRNAs (FImRNAs) were then determined by intersecting the upregulated DEGs with MulmRNAs, yielding 31 genes for miR-28–5p and 9 genes for miR-5683 and miR-145–3p. The lists of 31 genes associated with the first axis and 9 genes related to the second axis are provided in Supplementary Tables 3 and 4, respectively. By combining the components, a ceRNA network was formed, which was visualized in Cytoscape (Fig. 4).

### 3.4 Construction of PPI network and indentification of hub genes

**Fig. 5:**
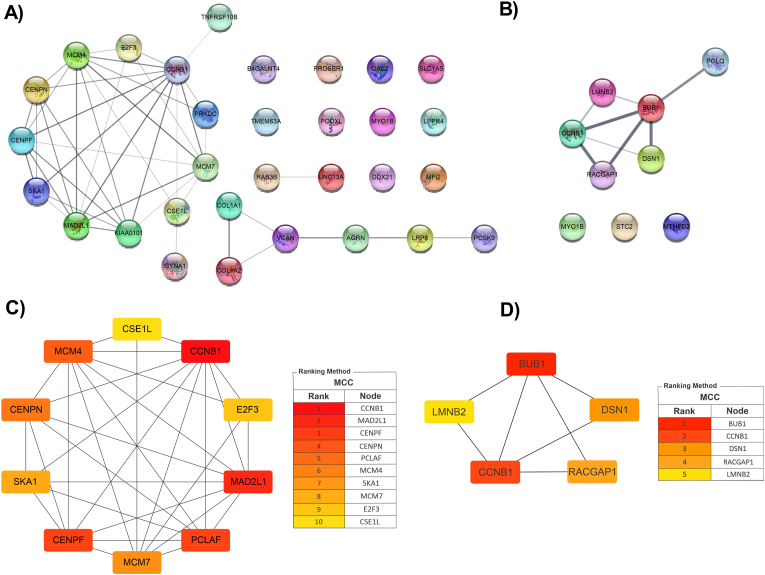
PPI analysis. The PPI networks of FImRNAs associated with hsa_circ_0043256 (**A**) and hsa_circ_0004789 (**B**) are shown. Genes that have no relationships with others are placed outside the network. Hub gene networks of the first (**C**) and second axis (**D**) are also shown. The importance of hub genes is determined based on a color scale, with red indicating higher importance and yellow indicating lower importance.

PPI networks were constructed using the STRING database to evaluate the biological interactions of genes that were visualized in Cytoscape (Fig. 5A and B). Also, the CytoHubba plugin in Cytoscape was used to detect hub genes using CytoHubba's MCC (Maximal Clique Centrality) ranking. The top 10 hub genes of circ_0043256 axis were *CSE1L*, *MCM4*, *CENPN*, *SKA1*, *CENPF*, *MCM7*, *KIAA0101 (PCLAF)*, *MAD2L1*, *E2F3* and *CCNB1* (Fig. 5C). The list of top five hub genes for circ_0004789 axis were *BUB1*, *LMNB2*, *CCNB1*, *RACGAP1* and *DSN1* (Fig. 5D). *CCNB1* was the common hub gene in two axes and was chosen for further analysis due to its importance in the promotion of cell cycle. A DEC-FImiRNA-hub gene network was constructed, including two circRNAs (circ_0043256 and circ_0004789), two miRNAs (miR-28–5p and miR-5683), and *CCNB1* as the hub gene, which were subjected to experimental evaluation. miR-5683 was selected for the second axis due to its larger |log2FoldChange|.

### 3.5 Functional enrichment analysis of DEGs

**Fig. 6:**
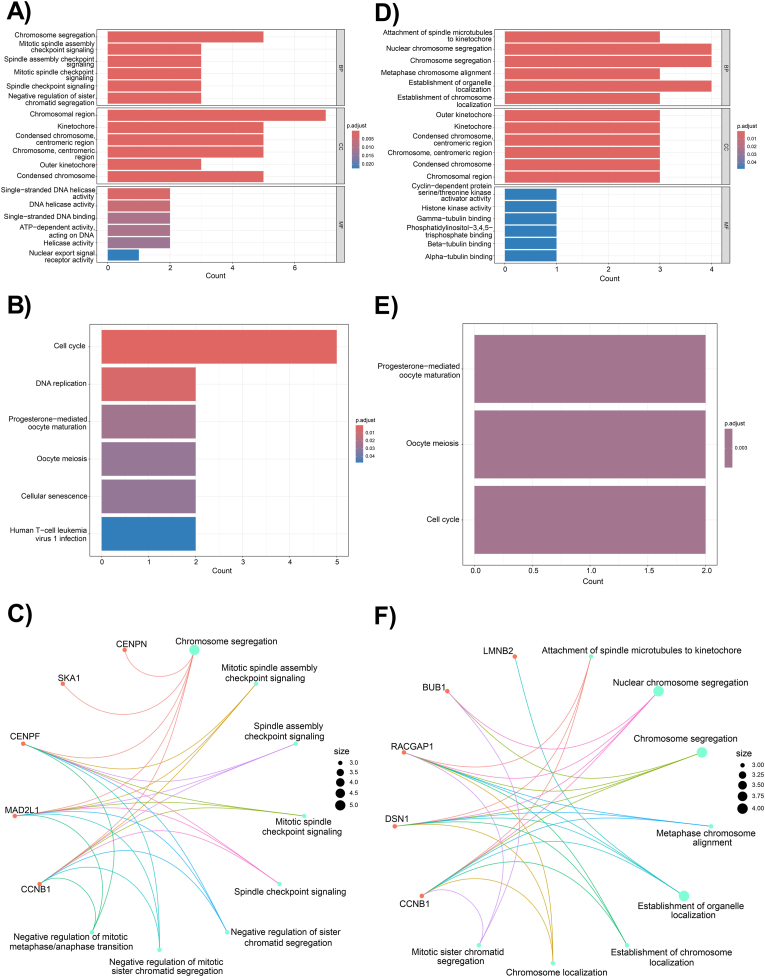
Functional enrichment analysis. The results of GO analysis for hsa_circ_0043256 (**A**) and hsa_circ_0004789 (**D**) are demonstrated in bar plots, which include three categories: biological process (BP), cellular component (CC), and molecular function (MF). The results of KEGG analysis are shown as bar plots for hsa_circ_0043256 (**B**) and hsa_circ_0004789 (**E**). A guide is located adjacent to each bar plot, indicating that greater red color intensity is associated with smaller adjusted p-values. Cnetplots show the relationship between each individual protein and its function in the BP category of GO analysis for hsa_circ_0043256 (**C**) and hsa_circ_0004789 (**F**).

In order to further investigate the importance of FImRNAs, functional enrichment analysis was carried out by GO and KEGG for the top ten FImRNAs of the circ_0043256 axis and the top five FImRNAs of the circ_0004789 axis. GO analysis was done in three categories: BP, CC, and MF. In BP terms, the top ten FImRNAs of the circ_0043256 axis were associated with chromosome segregation and mitotic spindle assembly checkpoint signaling, based on **gene counts** and the degree of significance. In CC terms, FImRNAs of circ_0043256 axis were enriched in chromosomal region, kinetochore, condensed chromosome, and centromeric region. For MF terms, FImRNAs of the circ_0043256 axis were enriched in single-stranded DNA helicase activity (Fig. 6A). KEGG analysis of FImRNAs of the circ_0043256 axis showed that these FImRNAs were enriched in cell cycle and DNA replication processes (Fig. 6B).

In BP terms, the top five FImRNAs of the circ_0004789 axis were enriched in attachment of spindle microtubules to kinetochore, nuclear chromosome segregation, chromosome segregation, and metaphase chromosome alignment. In CC terms, FImRNAs of the circ_0004789 axis were associated with the outer kinetochore, kinetochore, condensed chromosome, and centromeric region. For MF terms, FImRNAs of the circ_0004789 axis were enriched in cyclin-dependent protein serine/threonine kinase activator activity, histone kinase activity, gamma-tubulin binding, and phosphatidylinositol-3,4,5-trisphosphate binding (Fig. 6D). Also, the results of KEGG analysis revealed that these FImRNAs were related to progesterone-mediated oocyte maturation, oocyte meiosis, and cell cycle (Fig. 6E). The top FImRNAs of each axis and their connections are shown as cnetplots (Fig. 6C–F).

### 3.6 The expression of circ_0043256, circ_0004789, miR-28–5p, miR-5683, and CCNB1 in GC and non-tumor margin tissues

**Fig. 7:**
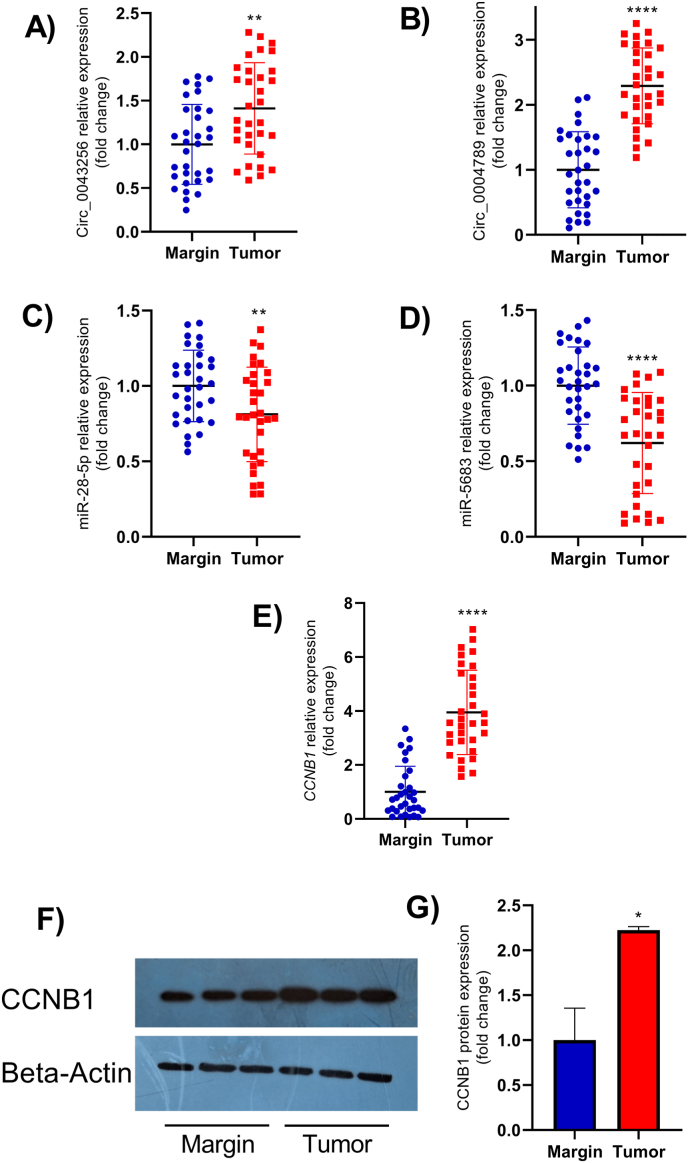
The expression levels of circ_0043256 (**A**), circ_0004789 (**B**), miR-28–5p (**C**), miR-5683 (**D**), and *CCNB1* (**E**) were evaluated by RT-qPCR in gastric cancer and non-tumor margin tissues. Representative Western blot bands (**F**) and quantitative densitometry measurement of CCNB1 protein expression (**G**) are also demonstrated. The uncropped Western blot images can be found in Supplementary Fig. 1. ∗p < 0.05, ∗∗p < 0.01, and ∗∗∗∗p < 0.0001.

We investigated the expression levels of circ_0043256, circ_0004789, miR-28–5p, miR-5683, and CCNB1 in 32 paired GC and non-tumor margin tissues by RT-qPCR. The expression levels of circ_0043256 and circ_0004789 were upregulated in GC samples relative to non-tumor margin tissues by 1.4 fold (p = 0.0014) and 2.3 fold (p < 0.0001), respectively (Fig. 7A and B). In contrast, the expression of miR-28–5p and miR-5683 was highly downregulated in tumor tissues, with miR-28–5p decreasing by 0.8 fold (p = 0.0087) and miR-5683 by 0.6 fold (p < 0.0001) (Fig. 7C and D). Additionally, the expression level of the CCNB1 gene, a prominent oncogene, was significantly increased in tumor tissues compared to non-tumor margin tissues (3.97-fold, p < 0.0001) (Fig. 7E). The CCNB1 protein level was also increased in tumor tissues (2.22-fold, p = 0.0257) (Fig. 7F and G). All of these results suggested that these two axes can play key roles in the pathogenesis of GC.

### 3.7 The correlation between the components of axes in GC tissues

**Fig. 8:**
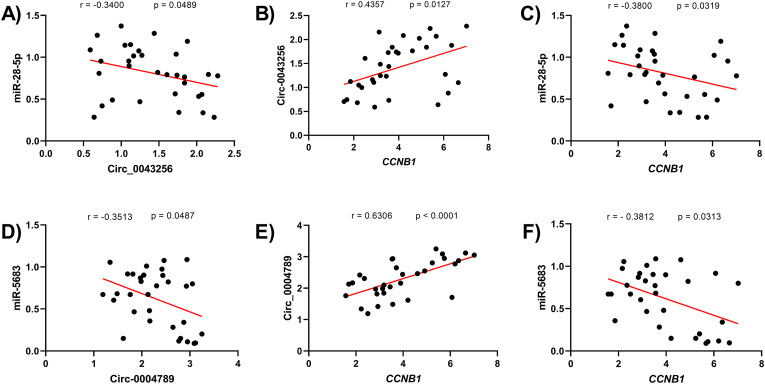
The correlation between the components of axes in GC tissues. Correlation analysis between hsa_circ_0043256 and miR-28–5p expression levels by Spearman correlation coefficient (**A**). Correlation analysis between *CCNB1* and hsa_circ_0043256 expression levels through Pearson method (**B**). Correlation analysis between *CCNB1* and miR-28–5p expression levels (Spearman) (**C**). Correlation analysis between hsa_circ_0004789 and miR-5683 expression levels by using Spearman correlation analysis (**D**). Correlation analysis between *CCNB1* and hsa_circ_0004789 expression levels through Pearson test (**E**). Correlation analysis between *CCNB1* and miR-5683 expression levels (Spearman) (**F**).

We examined the correlation between the components of the circ_0043256/miR-28–5p/*CCNB1* and circ_0004789/miR-5683/*CCNB1* axes in GC tissues. A negative correlation between circ_0043256 and miR-28–5p was observed with r = −0.3400 and p = 0.0489 (Fig. 8A). The correlation of circ_0043256 and *CCNB1* expression levels was positive (r = 0.4357 and p = 0.0127) (Fig. 8B). Moreover, the correlation between miR-28–5p and *CCNB1* mRNA levels was investigated. A negative correlation was found between them (r = −0.3800, p = 0.0319) (Fig. 8C).

A negative correlation was observed between circ_0004789 and miR-5683 expression levels with r = −0.3513, which was statistically confirmed (p = 0.0487) (Fig. 8D). The analysis demonstrated a positive correlation between circ_0004789 and *CCNB1* levels (r = 0.6306, p < 0.0001) (Fig. 8E). Finally, the correlation between the levels of miR-5683 and *CCNB1* was analyzed, revealing a negative relationship (r = −0.3812,p = 0.0313) (Fig. 8F).

### 3.8 ROC curve analysis of axes components

**Fig. 9:**
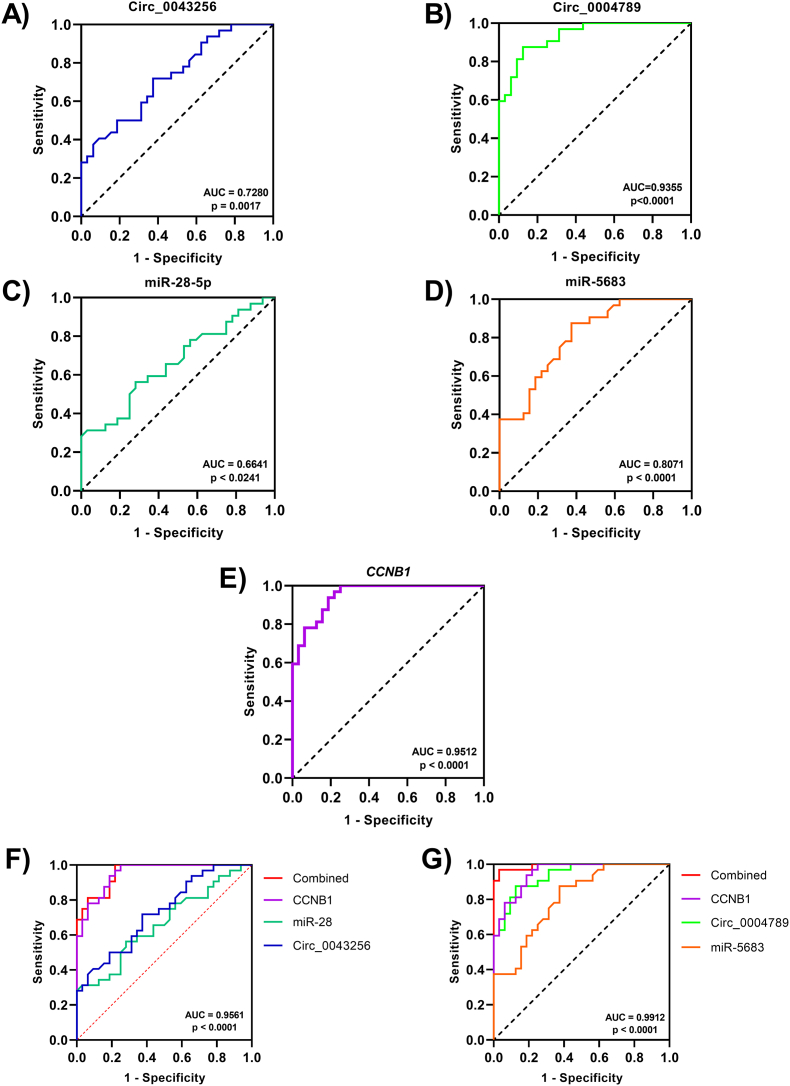
ROC curve analysis of circ_0043256 (**A**), circ_0004789 (**B**), miR-28–5p (**C**), miR-5683 (**D**), *CCNB1* (**E**), the combination of hsa_circ_0043256/miR-28–5p/*CCNB1* (**F**), and the combination of hsa_circ_0004789/miR-5683/*CCNB1* (**G**).

ROC curve analysis was performed to check the diagnostic value of the components of these two axes. Results showed the area under the curve (AUC) of circ_0043256, circ_0004789, miR-28–5p, miR-5683, and CCNB1were 0.7280 (p = 0.0017), 0.9355 (p < 0.0001), 0.6641 (p = 0.0241), 0.8071 (p < 0.0001), and 0.9512 (p < 0.0001), respectively (Fig. 9A–E).

Furthermore, we drew a combined ROC curve for both axes to determine the diagnostic value of all components of each axis. The AUC of the first axis, circ_0043256/miR-28–5p/CCNB1, was 0.9561 (p < 0.0001) (Fig. 9F). The AUC for the combined ROC curve of the second axis (circ_0004789/miR-5683/CCNB1) was higher, at 0.9912 (p < 0.0001) (Fig. 9G).

### 3.9 Investigating the relationship between the axes components with clinicopathological features of GC patients

This study included 32 patients, comprising 9 women and 23 men. The average age of patients was 63, ranging from 38 to 83 years. The average tumor size was 5 cm, and most patients, about 59.37 %, had larger tumors. Notably, the majority of patients (56.25 %) were in histological grade III + IV. The results of the correlation analysis between demographic characteristics and the expression levels of components of both axes are shown in Table 3, Table 4. It was found that circ_0043256 was related to lymphatic invasion (p = 0.040). Circ_0004789 was correlated with histological grade (p = 0.033), lymphatic invasion (p = 0.014), perineural invasion (p = 0.022), and lymph node involvement (N) (P = 0.033). *CCNB1* was associated with several pathological features, including histological grade (p = 0.011), lymphatic invasion (p = 0.024), perineural invasion (p = 0.040), and N (p = 0.023). miR-28–5p and miR-5683 were associated with N (p = 0.01) and tumor size (p = 0.016), respectively.

**Table 3:** Association of circ_0043256, circ_0004789, and *CCNB1* expression levels with GC clinico-pathological features.

Characteristics	Number n (%)	Expression of circ_0004789		χ2 p value	Expression of circ_0043256		χ2 p value	Expression of *CCNB1*		χ2 p value
		Low expression n (%)	High expression n (%)		Low expression n (%)	High expression n (%)		Low expression n (%)	High expression n (%)	
**Age (years)**				0.508 0.476			1.814 0.178			0.161 0.688
<63	14 (43.75)	8 (25.0)	6 (18.8)		6 (18.8)	8 (25.0)		8 (25.0)	6 (18.8)	
≥63	18 (56.25)	8 (25.0)	10 (31.3)		12 (37.5)	6 (18.8)		9 (28.1)	9 (28.1)	
**Gender**				1.391 0.238			0.002 0.960			0.922 0.337
Female	9 (28.12)	6 (18.8)	3 (9.4)		5 (15.6)	4 (12.5)		6 (18.8)	3 (9.4)	
Male	23 (71.87)	10 (31.3)	13 (40.6)		13 (40.6)	10 (31.3)		11 (34.4)	12 (37.5)	
**Site of primary**				2.672 0.263			1.131 0.568			0.077 0.962
Gastric Cardia	7 (21.87)	4 (12.5)	3 (9.4)		3 (9.4)	4 (12.5)		4 (12.5)	3 (9.4)	
Antrum	8 (25.0)	2 (6.3)	6 (18.8)		4 (12.5)	4 (12.5)		4 (12.5)	4 (12.5)	
Stomach	17 (53.12)	10 (31.3)	7 (21.9)		11 (34.4)	6 (18.8)		9 (28.1)	8 (25.0)	
**Tumor size (cm)**				0.130 0.719			0.907 0.341			0.427 0.513
<5	13 (40.62)	6 (18.8)	7 (21.9)		6 (18.8)	7 (21.9)		6 (18.8)	7 (21.9)	
≥5	19 (59.37)	10 (31.3)	9 (28.1)		12 (37.5)	7 (21.9)		11 (34.4)	8 (25.0)	
**Histological grade**				**4.57** **0.03∗**			0.653 0.419			**6.472** **0.011∗**
I + II	14 (43.75)	10 (31.3)	4 (12.5)		9 (28.1)	5 (15.6)		11 (34.4)	3 (9.4)	
III + IV	18 (56.25)	6 (18.8)	12 (37.5)		9 (28.1)	9 (28.1)		6 (18.8)	12 (37.5)	
**Lymphatic invasion**				**6.000** **0.01∗**			**4.233** **0.040∗**			**5.061** **0.024∗**
Yes	24 (75.0)	9 (28.1)	15 (46.9)		11 (34.4)	13 (40.6)		10 (31.3)	14 (43.75)	
No	8 (25.0)	7 (21.9)	1 (3.1)		7 (21.9)	1 (3.1)		7 (21.9)	1 (3.1)	
**Perineural invasion**				**5.236** **0.02∗**			1.117 0.290			**4.219** **0.040∗**
Yes	22 (68.75)	8 (25.0)	14 (43.8)		11 (34.4)	11 (34.4)		9 (28.1)	13 (40.6)	
No	10 (31.25)	8 (25.0)	2 (6.3)		7 (21.9)	3 (9.4)		8 (25.0)	2 (6.3)	
**Extracapsular nodal extention**				0.183 0.669			0.653 0.419			0.379 0.536
Yes	7(21.9)	3 (9.4) 13 (40.6)	4 (12.5)		3 (9.4)	4 (12.5)		3 (9.4)	4 (12.5)	
No	25 (78.2)		12 (37.5)		15 (46.9)	10 (31.3)		14 (43.8)	11 (34.4)	
**T**				0.821 0.365			2.201 0.138			2.706 0.100
T1+T2	6 (18.75)	4 (12.5)	2 (6.3)		5 (15.6)	1 (3.1)		5 (15.6)	1 (3.1)	
T3+T4	26 (81.25)	12 (37.5)	14 (43.8)		13 (40.6)	13 (40.6)		12 (37.5)	14 (43.8)	
**N**				**4.571** **0.03∗**			1.814 0.718			**5.181** **0.023∗**
N0+N1+N2	28 (87.5)	16 (57.1)	12 (37.5)		17 (53.1)	11 (34.4)		11 (34.4)	17 (53.1)	
N3	4 (12.5)	0 (0.0)	4 (12.5)		1 (3.1)	3 (9.4)		0 (0.0)	4 (12.5)	
**TNM staging**				0.000 1.000			0.847 0.358			1.414 0.234
I + II	12 (37.5)	6 (18.8)	6 (18.8)		8 (25.0)	4 (12.5)		8 (25.0)	4 (12.5)	
III + IV	20 (62.5)	10 (31.3)	10 (31.3)		10 (31.3)	10 (31.3)		9 (28.1)	11 (34.4)	
**Smoking**				0.000 1.000			0.073 0.788			0.878 0.349
Yes	4 (12.5)	2 (6.3)	2 (6.3)		2 (6.3)	2 (6.3)		3 (9.4)	1 (3.1)	
No	28 (87.5)	14 (43.8)	14 (43.8)		16 (50.0)	12 (37.5)		14 (43.8)	14 (43.8)	
**Family History**				0.508 0.476			0.395 0.530			0.161 0.688
Yes	14 (43.75)	6 (18.8)	8 (25.0)		7 (21.9)	7 (21.9)		8 (25.0)	6 (18.8)	
No	18 (56.25)	10 (31.3)	8 (25.0)		11 (34.4)	7 (21.9)		9 (28.1)	9 (28.1)	

Note: The gene expression data of the GC patients were divided into two categories of high expression and low expression based on the median. The chi-square test (χ2) was used to investigate the correlation between circ_0043256, circ_0004789, and *CCNB1* expressions and clinico-pathological features. ∗p value < 0.05.

**Table 4:** Association of miR-28–5p and miR-5683 expression levels with GC clinico-pathological features.

Characteristics	Expression of miR-5683			Expression of miR-28–5p		
	Low expression n (%)	High expression n (%)	χ2 p value	Low expression (%)	High expression n (%)	χ2 p value
**Age (years)**			0.395 0.530			0.051 0.821
<63	7 (21.9)	7 (21.9)		6 (18.8)	8 (25.0)	
≥63	7 (21.9)	11 (34.4)		7 (21.9)	11 (34.4)	
**Gender**			0.552 0.457			0.076 0.783
Female	3 (9.4)	6 (18.8)		4 (12.5)	5 (15.6)	
Male	11 (34.4)	12 (37.5)		9 (28.1)	14 (43.8)	
**Site of primary**			0.205 0.903			0.506 0.776
Gastric Cardia	3 (9.4)	4 (12.5)		3 (9.4)	4 (12.5)	
Antrum	3 (9.4)	5 (15.6)		4 (12.5)	4 (12.5)	
Stomach	8 (25.0)	9 (28.1)		6 (18.8)	11 (34.4)	
**Tumor size (cm)**			**5.776** **0.016∗**			0.277 0.598
<5	9 (28.1)	4 (12.5)		6 (18.8)	7 (21.9)	
≥5	5 (15.6)	14 (43.8)		7 (21.9)	12 (37.5)	
**Histological Grade**			2.330 0.127			3.802 0.051
I + II	4 (12.5)	10 (31.3)		3 (9.4)	11 (34.4)	
III + IV	10 (31.3)	8 (25.0)		10 (31.3)	8 (25.0)	
**Lymphatic invasion**			1.524 0.217			1.080 0.299
Yes	12 (37.5)	12 (37.5)		11 (34.4)	13 (40.6)	
No	2 (6.3)	6 (18.8)		2 (6.3)	6 (18.8)	
**Perineural invasion**			1.117 0.290			0.681 0.409
Yes	11 (34.4)	11 (34.4)		10 (31.3)	12 (37.5)	
No	3 (9.4)	7 (21.9)		3 (9.4)	7 (21.9)	
**Extracapsular nodal extension**			2.879 0.095			2.577 0.108
Yes	5 (15.6)	2 (6.3)		1 (3.1)	6 (18.8)	
No	9 (28.1)	16 (50.0)		12 (37.5)	13 (40.6)	
**T**			0.326 0.568			1.757 0.185
T1+T2	2 (6.3)	4 (12.5)		1 (3.1)	5 (15.6)	
T3+T4	12 (37.5)	14 (43.8)		12 (37.5)	14 (43.8)	
**N**			0.073 0.788			**6.681** **0.010∗**
N0+N1+N2	12 (37.5)	16 (50.0)		9 (28.1)	19 (59.4)	
N3	2 (6.3)	2 (6.3)		4 (12.5)	0 (0.0)	
**TNM staging**			0.034 0.854			1.943 0.163
I + II	5 (15.6)	7 (21.9)		3 (9.4)	9 (28.1)	
III + IV	9 (28.1)	11 (34.4)		10 (31.3)	10 (31.3)	
**Smoking**			0.653 0.419			0.463 0.496
Yes	13 (40.6)	15 (46.9)		12 (37.5)	16 (50.0)	
No	1 (3.1)	3 (9.4)		1 (3.1)	3 (9.4)	
**Family History**			0.653 0.419			0.051 0.821
Yes	5 (15.6)	9 (28.1)		6 (18.8)	8 (25.0)	
No	9 (28.1)	9 (28.1)		7 (21.9)	11 (34.4)	

Note: The gene expression data of the GC patients were divided into two categories of high expression and low expression based on the median. The chi-square test (χ2) was used to investigate the correlation between miR-28–5p and miR-5683 expressions and clinico-pathological features. ∗p value < 0.05.

### 3.10 Survival analysis of axes components

**Fig. 10:**
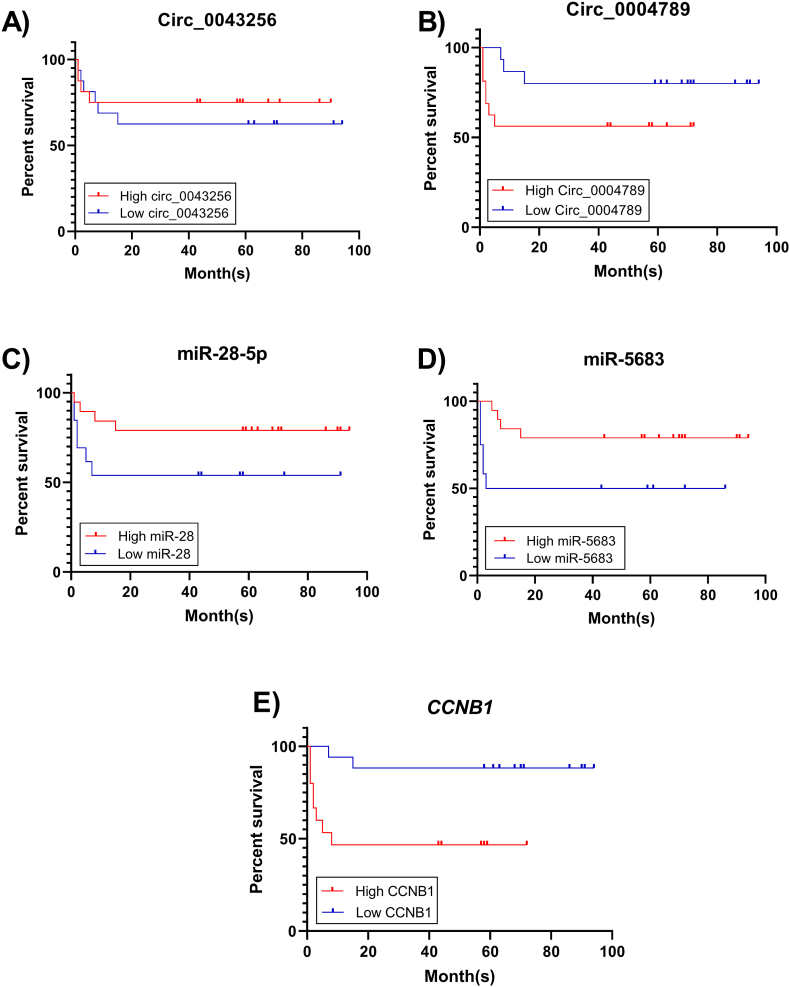
Overall survival curves of circ_0043256 (**A**), circ_0004789 (**B**), miR-28–5p (**C**), miR-5683 (**D**), and *CCNB1* (**E**) in GC patients, evaluated by Kaplan–Meier analysis.

Kaplan-Meier survival analysis was used to examine the potential of the axis components as predictors of GC patient outcomes. At first, the samples were divided into high- and low-expression groups based on the median expression level of each gene. *CCNB1* and miR-5683 were associated with the survival of GC patients, as confirmed by statistical analysis. GC patients with high expression of *CCNB1* had a lower survival rate than patients with low levels of this gene (p = 0.0067) (Fig. 10E). Conversely, patients with high levels of the miR-5683 had higher survival rates than those with lower expression (p = 0.0422) (Fig. 10D). Circ_0043256 did not show statistically significant results (p = 0.1015) (Fig. 10A). Meanwhile, GC patients with high expression levels of circ_0004789 had lower survival rates than those with low expression group, but this was not statistically significant (Fig. 10B). Additionally, high miR-28–5p expression level was associated with higher survival rate, but this was not statistically confirmed (p = 0.1030) (Fig. 10C), likely due to the small sample size.

## 4 Discussion

Despite all the efforts to understand the mechanisms underlying the development and treatment of GC, some molecular aspects of this disease remain unknown. CircRNAs, a group of ncRNAs, have received significant attention recently. However, many aspects of their functional mechanisms remain dark and unknown. The ceRNA hypothesis introduced circRNAs as miRNAs sponges, competing with other ncRNAs to absorb miRNAs and thereby prevent them from acting on target mRNAs.

We analyzed two GEO datasets and identified two exonic circRNAs, hsa_circ_0043256 and hsa_circ_0004789, that had not been previously studied in GC. Both were consistently upregulated in tumor tissues compared with non-tumor tissues, as confirmed by bioinformatics and RT-qPCR-based tissue validation. Studies conducted on circ_0043256 reported different results. For instance, studies of lung adenocarcinoma [24] and small hepatocellular carcinoma [25] reported upregulation of circ_0043256, whereas a pan-cancer study suggested reduced levels across several cancers, including GC [26]. In non-small cell lung cancer (NSCLC), Li et al. predicted decreased expression of circ_0043256 and proposed its diagnostic potential [27]. Interestingly, Yang et al. found downregulation by bioinformatics analysis but observed increased expression by RT-qPCR in NSCLC tumor cells [28]. These discrepancies may be due to the context-dependent functional roles of circ_0043256 across various cancers, underscoring the need for further mechanistic investigation. Few studies have been conducted on circ_0004789. Circ_0004789 is upregulated in papillary thyroid cancer(PTC) tissues and tumor cell lines. Circ_0004789 promotes the growth and development of PTC not through the ncRNAs axis but by direct interaction with CTNNBIP1, which disrupts its interaction with β-catenin and leads to the activation of the Wnt signaling pathway [29].

According to the ceRNA hypothesis, oncogenic circRNAs reduce the levels of target miRNAs. We predicted target miRNAs by intersecting CSCDmiRs with downregulated DEMIs, finding miR-28–5p for circ_0043256, and miR-5683 and miR-145–3p for circ_0004789. Based on the log_2_FoldChange value, miR-5683 was selected as the target for circ_0004789. RT-qPCR confirmed significant downregulation of miR-28–5p and miR-5683 in GC tissues, in agreement with their established tumor-suppressive roles. Low expression of miR-28–5p has been observed in GC tissues and cell lines, where it functions as a tumor suppressor by inhibiting migration and invasion. It has been proposed as a diagnostic biomarker [30,31]. Similarly, Miao et al. reported decreased miR-5683 expression in GC, showing that it suppresses proliferation by targeting PDK4 and mediates inhibition of glycolysis [32]. Another study demonstrated that miR-5683 interacts with HMGB1, contributing to the tumor-suppressive effect of Aloin in GC cells [33].

In the next step, potential target genes were predicted by intersecting upregulated DEGs and MulmRNAs. A regulatory ceRNA network was predicted from DECs-FImiRs and FImiRs-FImRNAs. Then, we constructed a PPI network for the target genes of two axes and identified the most important genes as hub genes. Among these, *CCNB1* was identified as a crucial hub gene in both axes and selected for further studies. CCNB1, a key effector of the cell cycle, forms a complex with a serine-threonine kinase enzyme (CDK1). The activated CCNB1-CDK1 complex initiates several steps in mitosis, including chromosome condensation, spindle formation, and nuclear membrane breakdown [34]. Elevated levels of CCNB1 are associated with tumor cell immortality and chromosomal instability, leading to tumor cell invasion and poor prognosis in cancer patients [34]. We measured the mRNA and protein levels of CCNB1 and found an increase, similar to other studies. The enrichment analysis results of top FImRNAs also indicated the importance of their functional role in cell cycle progression, particularly in chromosome segragation and mitosis. KEGG pathway analysis further confirmed that these genes are involved in the advancement of the cell cycle. These results suggest that circ_0043256 and circ_0004789 may contribute to tumorigenesis and GC tumor development through the circRNA/miRNA/mRNA axis. Although the GO and KEGG results showed that the predicted FImRNAs are mainly enriched in cell cycle and proliferation, based on multiple studies, these genes improve cancer cell growth through activation of signaling pathways like Wnt, PI3K/Akt, STAT3, and MAPK pathways [[35], [36], [37], [38], [39], [40], [41], [42]].

Finally, two novel ceRNA axes were predicted sharing the oncogenic target CCNB1: circ_0043256/miR-28–5p/CCNB1 and circ-0004789/miR-5683/CCNB1. Several additional axes have been proposed for these circRNAs, indicating their importance in cancer pathogenesis. In contrast to our findings, circ_0043256 has been reported as tumor-suppressive in lung cancer, acting through distinct axes (e.g., miR-1206/KLF2, miR-1252/ITCH, and multiple miRNA-mediated networks) to inhibit proliferation and growth [[43], [44], [45]]. Consistent with our findings, an earlier study suggested an oncogenic role for circ_0043256 in regulating the proliferation, migration, apoptosis, and glycolysis of GC cells via the miR-593–5p/RRM2 axis under CoCl_2_ induction [46]. Moreover, Zuo et al. predicted and validated the downregulation of both circRNAs in lung adenocarcinoma, implicating them in pathogenesis through the circ_0004789/miR-877–5p/C1orf115 and circ_0043256/miR-4496/PRICKLE2 axes [47]. The different roles of circ_0043256 in GC and lung cancer can be explained by several factors: differences in miRNA expression profiles, availability of MREs, the presence of regulatory factors such as RNA-binding proteins, and differences in the tumor microenvironment.

In our study, we investigated correlations between the axis components and reported negative correlations between circ_0043256 and miR-28–5p, and between circ-0004789 and miR-5683. Additionally, the correlation between miR-28–5p and *CCNB1 was* also investigated, and a negative relationship was confirmed; the same was true for miR-5683 and *CCNB1*. Interestingly, a positive correlation was observed between circ_0043256 and *CCNB1*, and also between circ_0004789 and *CCNB1*. The observed correlations among the expression levels of components in both axes were interestingly consistent with the ceRNA hypothesis; however, further confirmation through a dual luciferase reporter assay is needed to verify the interaction between the components in the two axes.

The ROC curve was drawn to evaluate the potential diagnostic value of the axis components in GC. The highest AUC was related to *CCNB1*, while the lowest was for miR-28–5p. Furthermore, the combined ROC curve of the circ-0004789/miR-5683/*CCNB1* axis had an AUC of 0.9912, indicating that the components of this axis, including circ_0004789, miR-5683, and *CCNB1*, can be effective in the diagnosis of GC. However, this needs to be investigated in a larger cohort in future studies.

To assess the prognostic relevance of the proposed axes, survival analysis was performed in GC patients. *CCNB1* and miR-5683 were associated with survival, with patients who had low *CCNB1* expression and high miR-5683 expression having longer survival, consistent with their predicted roles under the ceRNA hypothesis. Although the relationships between circ_0043256, circ_0004789, and miR-28–5p have not been statistically proven, there is a clear tendency for improved survival in patients with lower expression levels of circ_0004789 and higher expression of miR-28–5p. Future studies with larger sample sizes could be very useful. Previous studies similarly reported reduced survival in GC patients with miR-28–5p downregulation and CCNB1 positivity [30,48]. Furthermore, miR-5683 has been identified as an independent prognostic marker for overall survival in colon adenocarcinoma [49].

Examining the relationship between axis components and clinicopathological features, we found that high circ_0043256 expression is associated with lymphatic invasion. Elevated circ_0004789 and CCNB1 levels were observed in patients with histopathological grades III-IV, and correlated with lymphatic and perineural invasion. Additionally, circ_0004789 and CCNB1 showed an inverse association with N stage, being more highly expressed in N0–N2 groups. Previous studies have similarly linked CCNB1 expression in GC to regional lymph node metastasis [48,50]. miR-28–5p also correlated with N stage, with higher levels in N0–N2 compared to N3 patients. Xiao et al. reported reduced miR-28–5p expression in advanced TNM stage (IIIC-IV), T4 stage, and N3 lymph node status groups [30], while Jeddi et al. associated its downregulation with larger tumor size, higher grade, and metastasis [31]. For miR-5683, we observed higher expression in patients with larger tumors, although Miao et al. found the opposite, reporting reduced levels in larger GC tumors [32]. Our findings demonstrate that circ_0043256 and circ_0004789 are positively associated with deeper tumor invasion (T3/T4), advanced TNM stage, and lymph-node metastasis, supporting their potential oncogenic roles in GC. CCNB1, a predicted common target linked to poorer survival, showed a positive correlation with circRNA expression, suggesting a regulatory model in which circ_0043256 and circ_0004789 inhibit miR-28–5p and miR-5683, thereby upregulating CCNB1 and promoting cell cycle progression, tumor growth, and metastasis. Overall, this study evaluates the expression of circ_0043256 and circ_0004789 in GC tissues using integrated bioinformatics and tissue validation, demonstrating their diagnostic potential via ROC analyses and significant correlations with histopathological features and survival. A graphical summary of study steps is provided in the Graphical Abstract.

Our study had several limitations that should be addressed in future studies. Although the expression patterns of the predicted axis components and their correlations were supportive of the ceRNA hypothesis, their interactions were not investigated experimentally. It is suggested that validation of the axis component interaction using luciferase reporter assays, RNA pull-down, and immunoprecipitation be performed in future studies. Further *in vitro* cellular experiments and *in vivo* animal studies, along with functional experiments (knockdown/overexpression), are required to understand better the direct effects of circ_0043256 and circ_0004789 on GC development. Additionally, measuring the levels of these two circRNAs in larger sample sizes and in serum/plasma samples can help assess their potential as biomarkers in GC diagnosis and prognosis.

## 5 Conclusion

In this study, it was revealed that the expression of circ_0043256 and circ_0004789 increases in GC. Also, this study predicted two regulatory axes related to circ_0043256 and circ_0004789 with a common target mRNA: circ_0043256/miR-28–5p/CCNB1 and circ_0004789/miR-5683/CCNB1. Further investigation into these two new axes can clarify their potential as biomarkers for diagnosis, prognosis, and treatment of GC.

## Data availability statement

The RNA sequencing data used in the present study came from the publicly accessible TCGA-STAD project (https://portal.gdc.cancer.gov/projects/TCGA-STAD). Additionally, the microarray data were obtained from the GSE93541 and GSE78092 datasets available in the GEO database (https://www.ncbi.nlm.nih.gov/geo/). All the data of this study are included in this article and its supplementary data.

## Ethics approval and consent to participate

The ethical considerations of this study have been approved by ethics committee at Hamadan University of Medical Sciences (Ethical code: IR.UMSHA.REC.1401.749). The study described has been conducted in line with the Declaration of Helsinki for experiments involving humans. Informed consent was obtained for experimentation with human samples. The privacy rights of human subjects have been observed.

## Funding

This study was done with the financial support of the 10.13039/501100004697Hamadan University of Medical Sciences (Grant Number: 140110279168).

## CRediT authorship contribution statement

**Somayeh Aslani:** Conceptualization, Investigation, Methodology, Writing – original draft. **Ashkan Kalantary-Charvadeh:** Methodology, Writing – review & editing. **Pejman Morovat:** Data curation, Investigation. **Roghayeh Abbasalipourkabir:** Writing – review & editing. **Issa Nourmohammadi:** Writing – review & editing. **Amirnader Emami Razavi:** Methodology, Writing – review & editing. **Nasrin Ziamajidi:** Project administration, Supervision, Validation, Writing – review & editing.

## Declaration of competing interest

The authors declare that they have no known competing financial interests or personal relationships that could have appeared to influence the work reported in this paper.

## Data Availability

The RNA sequencing and the microarray data came from the publicly accessible TCGA-STAD project (https://portal.gdc.cancer.gov/projects/TCGA-STAD) and GEO database (https://www.ncbi.nlm.nih.gov/geo/).
