# Supplementary material for: Integrated bioinformatics and tissue-based validation reveal the oncogenic role of hsa_circ_0043256 and hsa_circ_0004789 in gastric cancer
Source: Biochem Biophys Rep. 2026 Jan 9;45:102442. doi: 10.1016/j.bbrep.2026.102442 (PMC12816855; doi:10.1016/j.bbrep.2026.102442)

Supplementary Table 1. Forward and reverse primers for all genes

| CCNB1 | F: GGTGACTTTGCTTTTGTGACTG |
| --- | --- |
|  | R: CGACATCAACCTCTCCAATCT |
| hsa_circ_0043256 | F: CGTATATGAAAGAGGAAGTGGA |
|  | R: CCAGCCAGCCAGTATCAA |
| hsa_circ_0004789 | F: ATCAACCGCCTCAAAGACA |
|  | R: AGATCCATCAACCACCACC |
| Beta actin | F: GAGCCTCGCCTTTGCCGATCC |
|  | R: ACATGCCGGAGCCGTTGTCG |
| hsa-miR-28-5p | RTprimer (specific): GTCGTATCCAGTGCAGGGTCCGAGGTATTCGCACTGGATACGACCTCAAT |
|  | F: GACAAGGAGCTCACAGTCTAT |
|  | R: GTCGTATCCAGTGCAGGGT |
| hsa-miR-5683 | RTprimer(specific): GTCGTATCCAGTGCAGGGTCCGAGGTATTCGCACTGGATACGACGAAGTC |
|  | F: AACCGGTACAGATGCAGATTCT |
|  | R: GTCGTATCCAGTGCAGGGT |
| U6 | RTprimer(specific): GTCGTATCCAGTGCAGGGTCCGAGGTATTCGCACTGGATACGACAAAATA |
|  | F: GTGCTCGCTTCGGCAGCA |
|  | R: GTCGTATCCAGTGCAGGGT |

Supplementary Table 2. 58 DECs with their essential features.

| **CircRNAs** | **Up/Down** | **logFC** | **adj.p.Val** | **Gene_ID** |
| --- | --- | --- | --- | --- |
| hsa_circ_0000220 | Up | 2.191260001 | 0.018119284 | ASCRP000940 |
| hsa_circ_0023642 | Up | 2.007722217 | 0.00745396 | ASCRP001261 |
| hsa_circ_0072263 | Up | 1.894836597 | 0.018552051 | ASCRP004104 |
| hsa_circ_0005252 | Up | 1.872084331 | 0.016723887 | ASCRP001771 |
| hsa_circ_0032664 | Up | 1.871447941 | 0.00745396 | ASCRP001763 |
| hsa_circ_0043256 | Up | 1.812552063 | 0.017439898 | ASCRP002381 |
| hsa_circ_0092367 | Up | 1.665562753 | 0.026541793 | ASCRP005323 |
| hsa_circ_0032683 | Up | 1.603459338 | 0.016723887 | ASCRP001766 |
| hsa_circ_0001748 | Up | 1.555734907 | 0.029423684 | ASCRP000370 |
| hsa_circ_0002300 | Up | 1.521875218 | 0.00745396 | ASCRP004304 |
| hsa_circ_0004552 | Up | 1.464805777 | 0.025362123 | ASCRP002764 |
| hsa_circ_0002019 | Up | 1.375726154 | 0.034540456 | ASCRP001355 |
| hsa_circ_0076092 | Up | 1.34518688 | 0.0425702 | ASCRP004372 |
| hsa_circ_0000788 | Up | 1.318256765 | 0.011825136 | ASCRP000144 |
| hsa_circ_0079385 | Up | 1.315468305 | 0.021610363 | ASCRP004574 |
| hsa_circ_0000979 | Up | 1.310884701 | 0.032886095 | ASCRP000302 |
| hsa_circ_0001013 | Up | 1.250821714 | 0.010452516 | ASCRP000098 |
| hsa_circ_0000632 | Up | 1.245767056 | 0.024781768 | ASCRP000137 |
| hsa_circ_0056856 | Up | 1.232466421 | 0.019526708 | ASCRP003146 |
| hsa_circ_0000958 | Up | 1.22531886 | 0.047716612 | ASCRP002913 |
| hsa_circ_0001184 | Up | 1.186739873 | 0.016723887 | ASCRP000150 |
| hsa_circ_0002881 | Up | 1.15880689 | 0.029052163 | ASCRP001368 |
| hsa_circ_0004789 | Up | 1.157292422 | 0.021610363 | ASCRP002503 |
| hsa_circ_0069249 | Up | 1.130988305 | 0.018386771 | ASCRP003903 |
| hsa_circ_0001221 | Up | 1.126466865 | 0.00745396 | ASCRP000094 |
| hsa_circ_0006110 | Up | 1.109337653 | 0.028000386 | ASCRP003040 |
| hsa_circ_0001936 | Up | 1.106339871 | 0.017355655 | ASCRP005260 |
| hsa_circ_0001693 | Up | 1.09830625 | 0.017355655 | ASCRP000379 |
| hsa_circ_0049271 | Up | 1.094975414 | 0.016723887 | ASCRP002761 |
| hsa_circ_0001887 | Up | 1.069143221 | 0.035049838 | ASCRP000172 |
| hsa_circ_0049249 | Up | 1.055341071 | 0.022769332 | ASCRP002760 |
| hsa_circ_0023064 | Up | 1.040575019 | 0.008633370 | ASCRP001233 |
| hsa_circ_0005139 | Up | 1.006897662 | 0.0425702 | ASCRP001732 |
| hsa_circ_0001955 | Down | -1.013353231 | 0.016723887 | ASCRP001906 |
| hsa_circ_0003892 | Down | -1.017025613 | 0.018386771 | ASCRP002770 |
| hsa_circ_0000981 | Down | -1.055634821 | 0.00760304 | ASCRP000286 |
| hsa_circ_0060063 | Down | -1.085351982 | 0.017439898 | ASCRP003345 |
| hsa_circ_0092363 | Down | -1.087882917 | 0.024987277 | ASCRP005320 |
| hsa_circ_0092342 | Down | -1.097954143 | 0.043144263 | ASCRP005315 |
| hsa_circ_0084501 | Down | -1.131529561 | 0.018119284 | ASCRP004878 |
| hsa_circ_0051258 | Down | -1.189965744 | 0.00745396 | ASCRP002875 |
| hsa_circ_0008618 | Down | -1.298333314 | 0.016866329 | ASCRP004048 |
| hsa_circ_0000993 | Down | -1.298465671 | 0.016723887 | ASCRP003006 |
| hsa_circ_0006014 | Down | -1.339260236 | 0.028866333 | ASCRP003263 |
| hsa_circ_0067934 | Down | -1.347678742 | 0.016866329 | ASCRP003808 |
| hsa_circ_0008016 | Down | -1.349833846 | 0.035112501 | ASCRP004848 |
| hsa_circ_0007991 | Down | -1.36225167 | 0.00745396 | ASCRP000474 |
| hsa_circ_0048607 | Down | -1.401458256 | 0.00745396 | ASCRP002736 |
| hsa_circ_0043438 | Down | -1.450025471 | 0.00745396 | ASCRP002396 |
| hsa_circ_0040039 | Down | -1.468174267 | 0.043165638 | ASCRP002195 |
| hsa_circ_0002965 | Down | -1.607968304 | 0.035112501 | ASCRP004950 |
| hsa_circ_0005051 | Down | -1.748656324 | 0.029522918 | ASCRP003857 |
| hsa_circ_0041732 | Down | -1.893170559 | 0.025738059 | ASCRP002307 |
| hsa_circ_0083766 | Down | -1.933654933 | 0.039632783 | ASCRP004828 |
| hsa_circ_0000511 | Down | -2.294371007 | 0.034540456 | ASCRP000385 |
| hsa_circ_0008139 | Down | -2.301259308 | 0.025738059 | ASCRP004673 |
| hsa_circ_0001190 | Down | -3.099974032 | 0.021071736 | ASCRP000339 |
| hsa_circ_0092337 | Down | -3.683110289 | 0.00745396 | ASCRP005325 |

Supplementary Table 3. 31 target mRNAs related to the first axis are listed along with their key characteristics.

| **Target_symbol** | **Target_ensembl** | **logFC** | **FDR** |
| --- | --- | --- | --- |
| AGRN | ENSG00000188157 | 1.57937245 | 2.66E-21 |
| B4GALNT4 | ENSG00000182272 | 1.836852482 | 6.50E-06 |
| CCNB1 | ENSG00000134057 | 1.364041088 | 6.51E-15 |
| CENPF | ENSG00000117724 | 2.270299056 | 1.81E-32 |
| CENPN | ENSG00000166451 | 1.11621396 | 5.26E-17 |
| COL1A1 | ENSG00000108821 | 2.983898441 | 2.55E-33 |
| COL1A2 | ENSG00000164692 | 1.884754497 | 2.42E-16 |
| CSE1L | ENSG00000124207 | 1.08834034 | 7.78E-18 |
| DDX21 | ENSG00000165732 | 1.068923442 | 2.65E-21 |
| E2F3 | ENSG00000112242 | 1.306610243 | 1.29E-33 |
| ISYNA1 | ENSG00000105655 | 1.068517664 | 7.84E-06 |
| LRP8 | ENSG00000157193 | 2.238438606 | 1.76E-24 |
| MAD2L1 | ENSG00000164109 | 1.725023211 | 9.20E-23 |
| MCM4 | ENSG00000104738 | 1.390636704 | 4.92E-19 |
| MCM7 | ENSG00000166508 | 1.247589669 | 1.78E-14 |
| MELTF | ENSG00000163975 | 2.916648115 | 2.39E-26 |
| MYO1B | ENSG00000128641 | 1.233601915 | 5.53E-18 |
| OAS2 | ENSG00000111335 | 1.064200574 | 1.21E-06 |
| PCLAF/KIAA0101 | ENSG00000166803 | 1.553576933 | 1.70E-16 |
| PCSK9 | ENSG00000169174 | 2.089295119 | 1.17E-10 |
| PLPPR4 | ENSG00000117600 | 1.402891778 | 1.26E-07 |
| PODXL | ENSG00000128567 | 1.253220313 | 1.67E-17 |
| PRKDC | ENSG00000253729 | 1.079632602 | 2.59E-17 |
| PROSER1 | ENSG00000120685 | 1.008031219 | 6.78E-16 |
| RAB3B | ENSG00000169213 | 1.069287539 | 0.001215 |
| SKA1 | ENSG00000154839 | 1.714142022 | 7.80E-20 |
| SLC1A5 | ENSG00000105281 | 1.153010322 | 1.29E-09 |
| TMEM63A | ENSG00000196187 | 1.026172567 | 2.21E-13 |
| TNFRSF10B | ENSG00000120889 | 1.327375895 | 4.79E-22 |
| UNC13A | ENSG00000130477 | 1.413544566 | 4.45E-05 |
| VCAN | ENSG00000038427 | 1.664074977 | 6.10E-14 |

FDR: false discovery rate

Supplementary Table 4. 9 target mRNAs related to the second axis are listed along with their key characteristics.

| **Target_symbol** | **Target_ensembl** | **logFC** | **FDR** | **miRNA** |
| --- | --- | --- | --- | --- |
| BUB1 | ENSG00000169679 | 2.00633620337143 | 2.72561927617715E-29 | miR-145-3p |
| CCNB1 | ENSG00000134057 | 1.36404108811184 | 6.50715532146397E-15 | miR-5683 |
| DSN1 | ENSG00000149636 | 1.04591141351031 | 1.89640158889472E-17 | miR-5683 |
| LMNB2 | ENSG00000176619 | 1.01191245329442 | 7.42419623797095E-14 | miR-145-3p |
| MTHFD2 | ENSG00000065911 | 1.15781421231259 | 5.69441552217203E-18 | miR-5683 |
| MYO1B | ENSG00000128641 | 1.23360191546974 | 5.53181405946684e-18 | miR-145-3p |
| POLQ | ENSG00000051341 | 1.91015327812468 | 1.77431843293823E-22 | miR-5683 |
| RACGAP1 | ENSG00000161800 | 1.0344330114137 | 8.59305140668077E-14 | miR-5683 |
| STC2 | ENSG00000113739 | 1.62928771156002 | 6.08398827042617E-12 | miR-5683 |

FDR: false discovery rate

**Supplementary Figure 1.** Original un-cropped western blot images used for data presented.

CCNB1-repeat 1


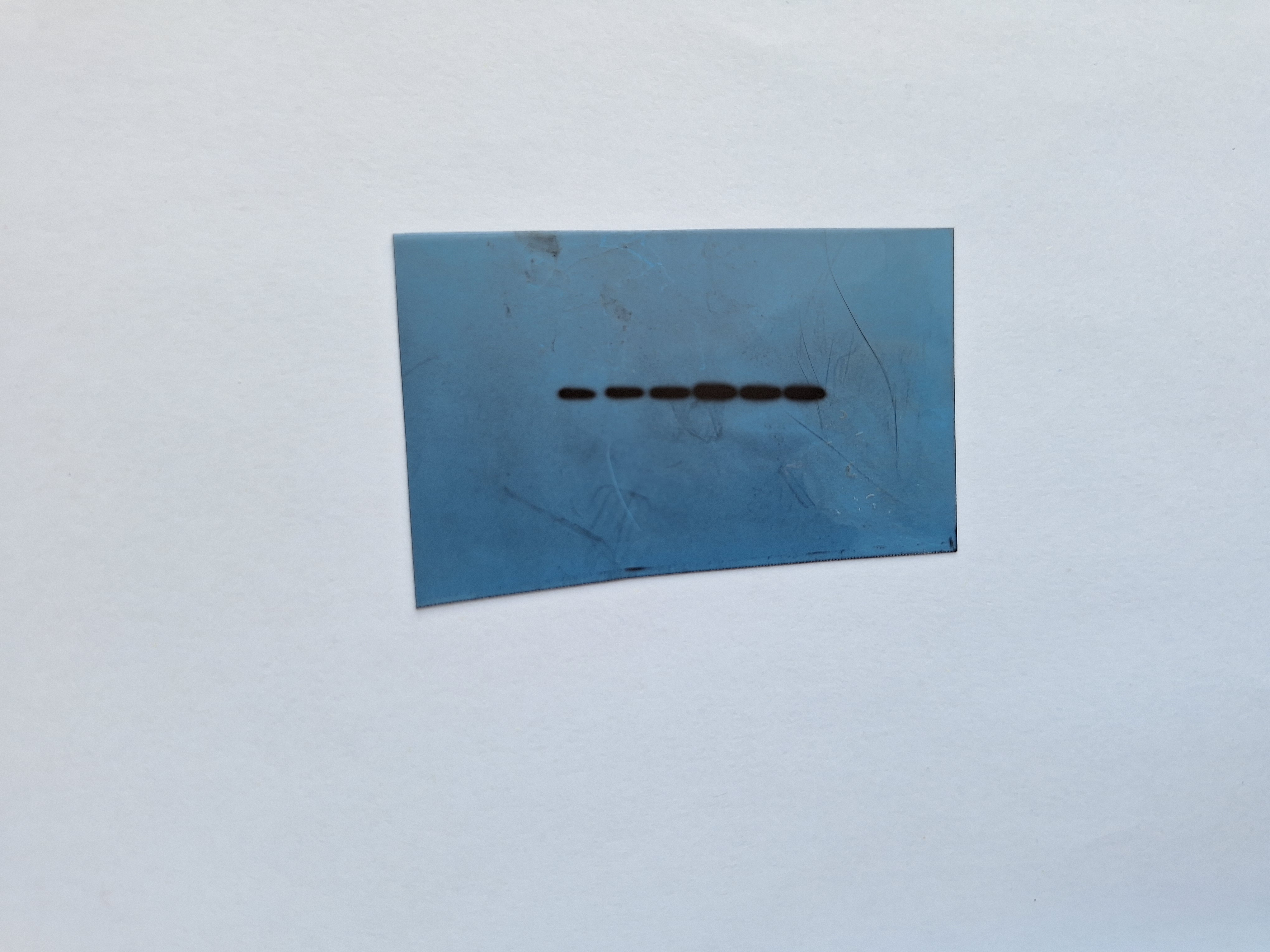


CCNB1-repeat 2


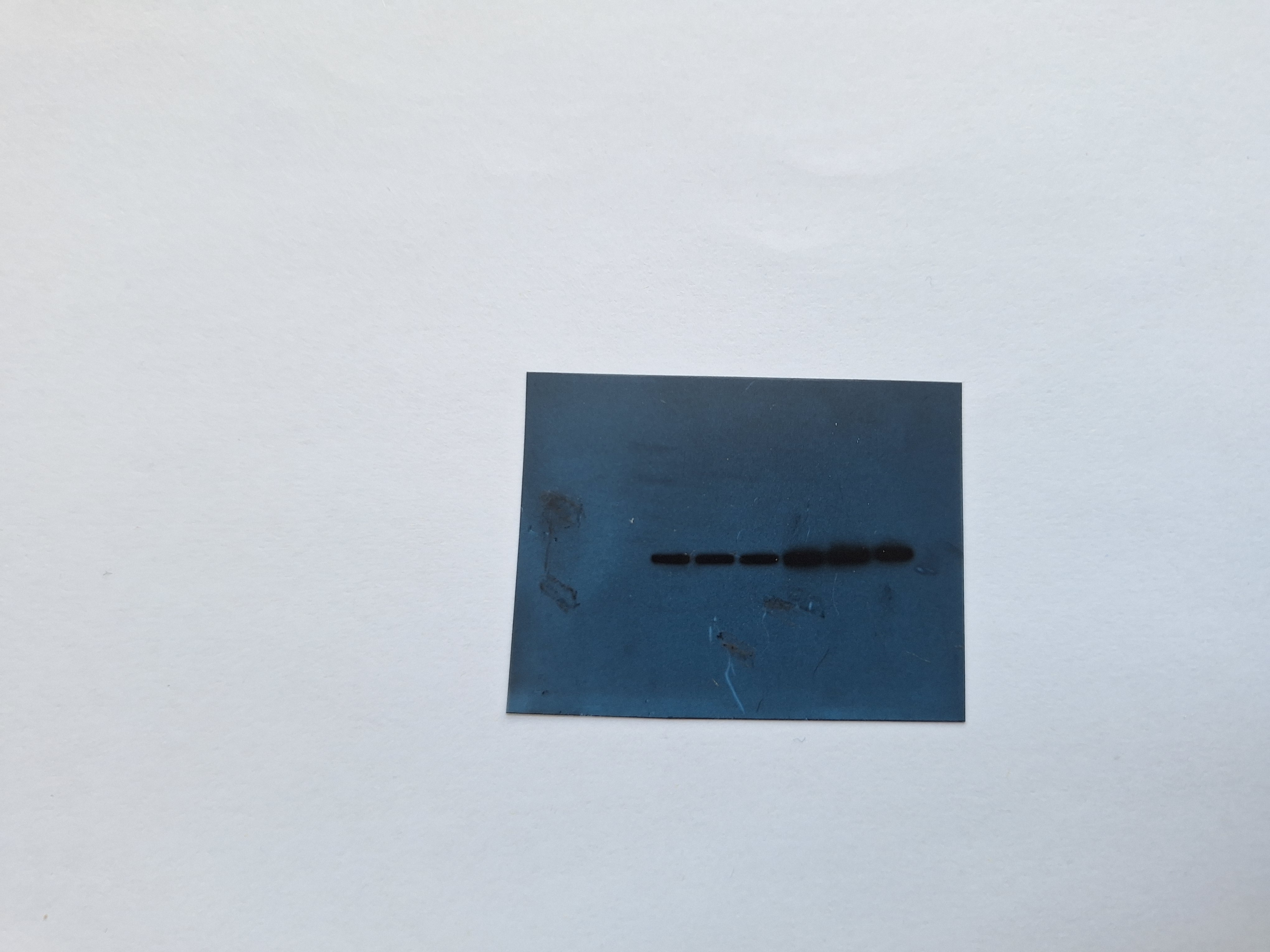


CCNB1-repeat3


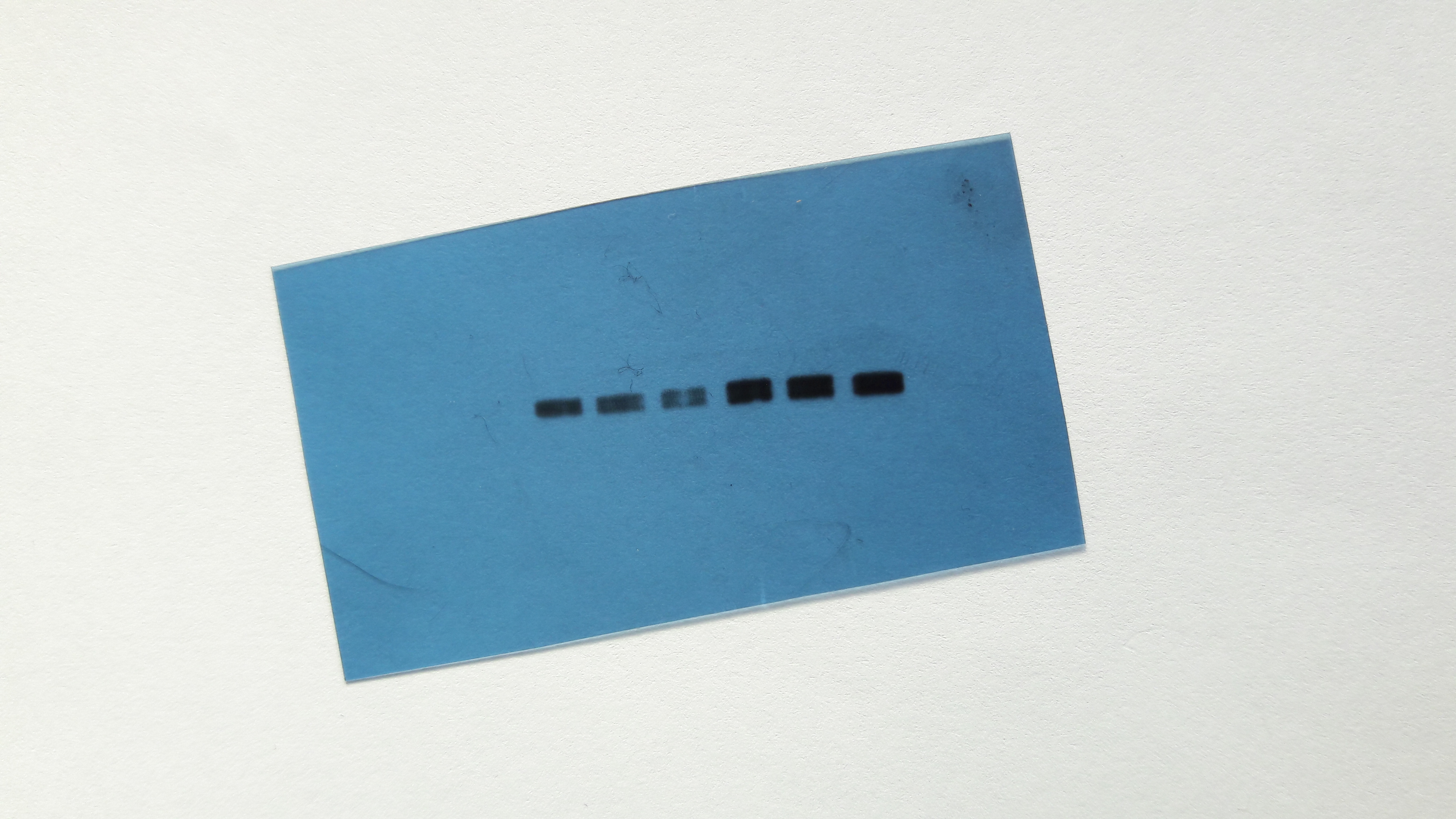


β-Actin- repeat 1
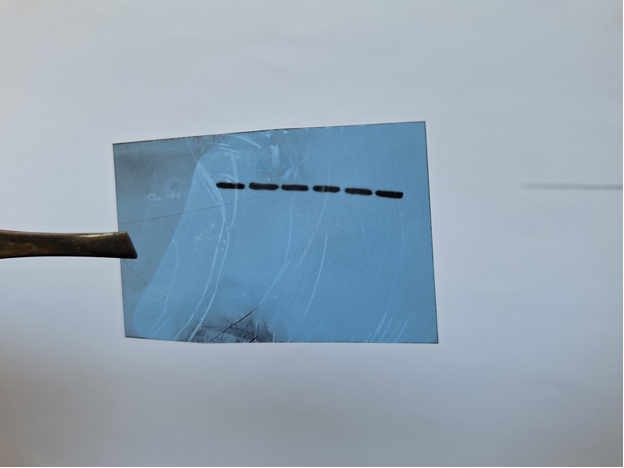


β-Actin- repeat 2


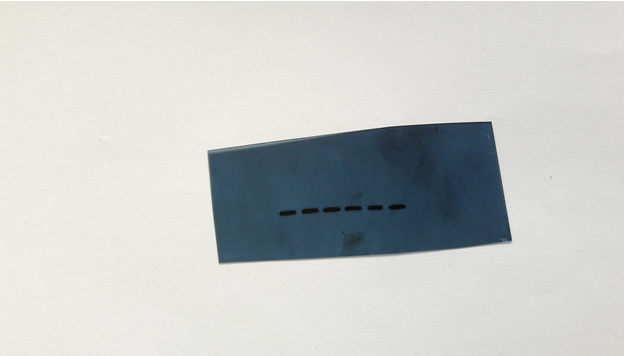


β-Actin- repeat 3


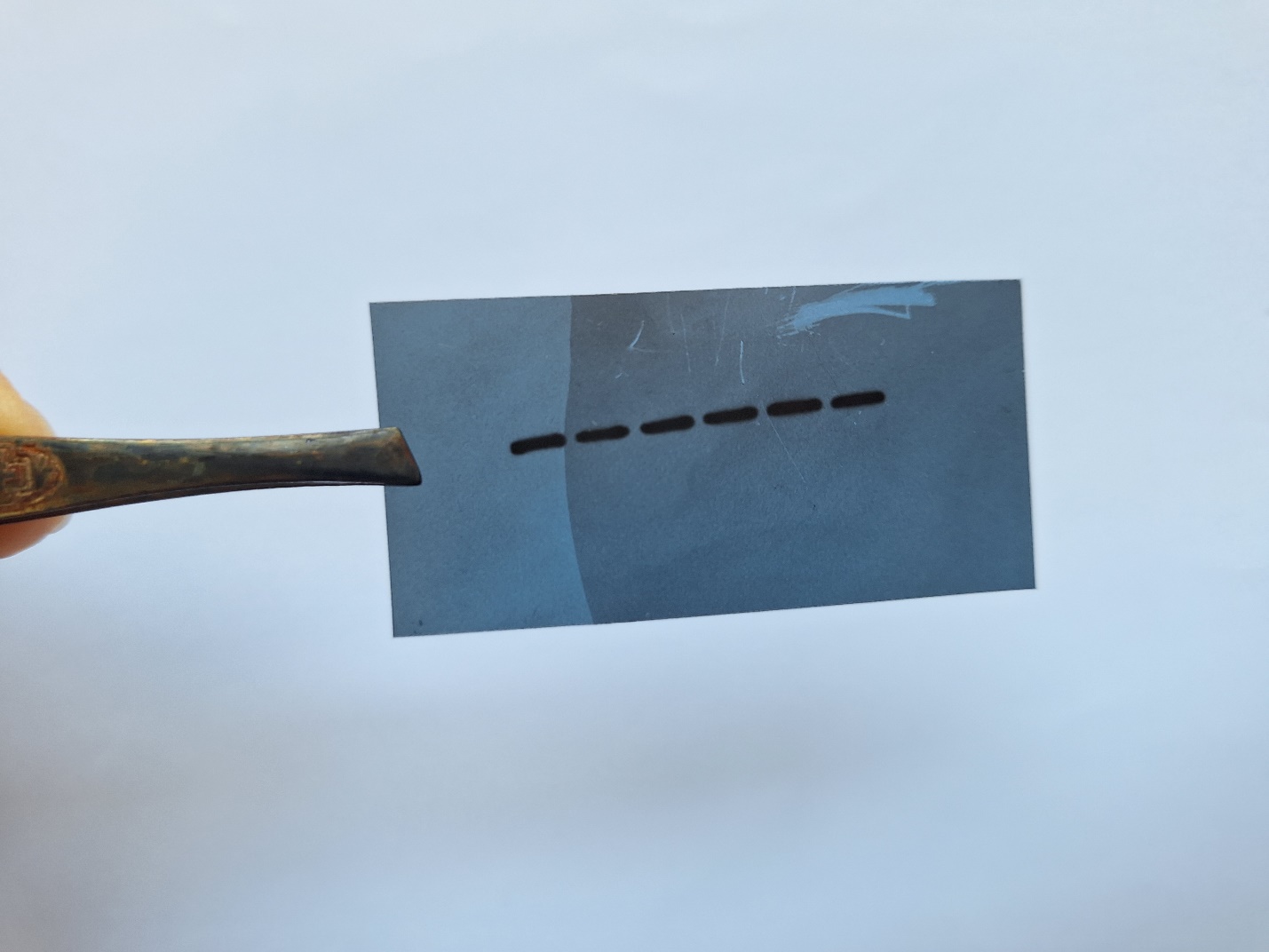

Supplement: Multimedia component 1 [file mmc1.docx]
